# Exploring the symptoms and psychological experiences among lung cancer convalescence patients after radical lobectomy: A qualitative study

**DOI:** 10.1002/cam4.70048

**Published:** 2024-07-31

**Authors:** Julan Xiao, Yueming Peng, Yun Li, FengQing Ye, Zhixong Zeng, XiaoXu Lin, Yanheng Xie, Sijiao Cheng, Yi Wen, Weixiang Luo

**Affiliations:** ^1^ Department of Thoracic Surgery Shenzhen People's Hospital (The Second Clinical Medical College, Jinan University; The First Affiliated Hospital, Southern University of Science and Technology) Shenzhen Guangdong China; ^2^ Shenzhen Clinical Research Centre for Geriatrics Shenzhen People's Hospital Shenzhen Guangdong China; ^3^ Department of Nursing Shenzhen People's Hospital (The Second Clinical Medical College, Jinan University, The First Affiliated Hospital; Southern University of Science and Technology) Shenzhen Guangdong China; ^4^ Department of the Operating Room Shenzhen People's Hospital (The Second Clinical Medical College, Jinan University; The First Affiliated Hospital, Southern University of Science and Technology) Shenzhen Guangdong China

**Keywords:** lung cancer, postoperative period, psychological feelings, qualitative study, rehabilitation nursing, symptom experiences

## Abstract

**Objective:**

This study aims to explore the symptom experiences and psychological feelings of lung cancer patients after radical lobectomy in China.

**Methods:**

A qualitative study was conducted using face‐to‐face semistructured interviews with lung cancer patients who had a radical lobectomy for treatment of their cancer during the convalescence period. Participants (*n* = 18) were recruited from a general hospital in China, and patients were selected using purposive sampling from September 2021 to February 2022. Interviews were recorded and transcribed verbatim, and Colaizzi's seven‐step method of phenomenology was used. The Consolidated Criteria for Reporting Qualitative Research (COREQ) checklist was conducted to report the study.

**Results:**

Four themes were extracted from the interview data: physiological dimensions (fatigue, cough or sputum, chest tightness and shortness of breath, daily activities affected, sleep disturbance, lack of appetite); psychological dimensions (negative emotion, fear of cancer recurrence, learning to accept reality, strengthened faith and hope); family dimensions (heavy economic burden, perceived family care, improved health management behavior); and social dimensions (perceived support of medical staff, decreased sense of social value and self‐identity, changes in social and working style).

**Conclusion:**

Lung cancer patients are still troubled by many problems during the postoperative recovery period. Medical staff should design and implement effective evaluations and targeted interventions for patients' physical and mental health as soon as possible to improve patients' physical and mental health, as well as their quality of life.

## 1 INTRODUCTION

Lung cancer is a type of malignant lung tumor that develops from bronchial mucosal epithelial cells; it remains the most common malignant tumor in the world, with the highest morbidity and mortality rates in China.^1^ In 2020, the number of new lung cancer cases was estimated to be 2.21 million, accounting for 11.4% of all new cancer cases; lung cancer remains the leading cause of cancer death, with an estimated 1.8 million deaths, accounting for 18% of all cancer deaths worldwide.^2^ The outcomes of lung cancer are among the poorest of all tumor types, with a 5‐year survival rate of approximately 10%–20% across all stages^3^; moreover, survivors are faced with the intense psychological pain due to disease stigma and fear of recurrence,^4^ which will exist across their lifetime. Compared to other cancers, lung cancer is associated with a greater symptom burden related to the disease and its treatment during the course of the illness.^5^ Globally, the cancer burden is projected to double by 2050, with lung cancer at the top of the list.^6^ This can greatly impact patients' quality of life (QOL) and functional ability, ultimately affecting their chances of survival. Lung cancer, as a negative life event, can cause psychological distress in patients, such as depression, anxiety, and sleep disorders.^7^

Surgical treatment, chemotherapy, radiotherapy, molecular targeted therapy, and immunotherapy are currently available to treat lung cancer.^8^ With the development and application of minimally invasive techniques in lung cancer treatment, surgery‐based treatment is still the preferred and main treatment modality for lung cancer.^9^ However, lung cancer surgery can have significant negative impacts on patients, who may experience a range of severe symptoms both during the postoperative period and even after being discharged from the hospital. Patients with lung cancer may experience various physical symptoms (e.g., pain, weakness, constipation, cough, shortness of breath, and sleep disturbance).^10^ In addition, due to the high risks related to surgery, surgical trauma and anesthesia frequently impart psychological and physiological impacts on the patient, causing a greater negative impact on the patient's endocrine, circulatory, and neurological systems.^11^ Surgical treatment is associated with numerous physical and psychological symptoms. These symptoms can lead to functional decline and poor QOL.^12^ Previous studies have shown that lung cancer patients have higher rates of psychosomatic symptoms as well as limited mental and role functioning, and have a poorer QOL than healthy people and other cancer patients.^13^ Therefore, more attention should be given to the symptoms and psychological status of lung cancer patients to improve their health‐related QOL.

Radical lobectomy is a common type of surgery for lung cancer, which refers to the complete and thorough surgical removal of the lobes of the lung at the site of the lung cancer lesion in order to achieve the radical nature of the surgery and at the same time to enhance local control and long‐term survival of lung cancer patients.^14^ Low‐dose spiral computed tomography (CT) has been increasingly used in early lung cancer screening and routine physical examinations in recent years, with an emphasis on cancer screening in China and the improvement of people's health awareness; this has led to an increase in the number of lung cancer surgery patients.^15^ As a result, more patients live as lung cancer survivors with thoracic surgery complications and loss of lung tissue and function. In addition, the physiological and emotional pain suffered by these patients increases manifold with disease progression. Regrettably, there has been a lack of research on the psychological and emotional impact of lung cancer after radical lobectomy.^5^

Additionally, as lung cancer patients navigate the long road to recovery following surgery, their symptoms and psychological well‐being can have a significant impact on their overall QOL due to the extended treatment period, poor prognosis, and high cost associated with their care.^16^, ^17^ At present, health care professionals tend to prioritize physiological indicators over the impact of patients' symptom distress and psychological well‐being on their QOL during the recovery process. Therefore, to fill these research gaps, a deeper understanding of the symptoms and psychological experience of lung cancer convalescent patients after radical lobectomy is needed; such an understanding may contribute to the development of targeted and effective strategies for enhancing lung cancer patient symptom management and continuity of care.

Qualitative research concentrates on the essence of an experience and enables people to be understood within their own culture and life.^18^ Therefore, the purpose of this qualitative study is to shed light on the symptom experiences and psychological feelings of lung cancer patients after undergoing a radical lobectomy. This study may help to identify the postoperative symptoms and emotional problems of patients with lung cancer, and provide suggestions for specific interventions to improve patients' physical and mental health, as well as their QOL.

## 2 METHODS

### 2.1 Design

This qualitative study, which used a phenomenological approach, was conducted following the Consolidated Criteria for Reporting Qualitative Research (COREQ) checklist.^19^ The phenomenological approach is a research strategy that aims to provide a comprehensive description of a particular phenomenon, as it relates to the experiences of those who have lived through it.^20^ In this study, we used in depth, open‐ended, semistructured, and face‐to‐face methods to explore the symptom experiences and psychological feelings of patients living with lung cancer after undergoing a radical lobectomy between September 2021 and February 2022.

### 2.2 Participants and recruitment

Participants were selected by purposive sampling; a total sample of 18 lung cancer patients that represented a diversity of age, gender, educational level, socioeconomic status, pathological type, and time since confirmed lung cancer diagnosis was established. The inclusion criteria included (1) patients who were histologically diagnosed with primary lung cancer and had completed radical surgery for lung cancer, and (2) patients aged 18 or older. The exclusion criteria were as follows: (1) patients with comorbidities, such as deafness and inability to communicate effectively, (2) patients with preexisting mental illness, and (3) patients with severe organ and cardiovascular dysfunction. Patients were selected from the thoracic surgery department of a 3000‐bed tertiary hospital in Shenzhen, China. Twenty participants were invited for our interviews, and two patients declined owing to lack of interest. In total, 18 patients with lung cancer participated in the study.

### 2.3 Procedures and data collection

The interviews were conducted by a full‐time nurse (as well as the first researcher, Julan Xiao) who had both a master's degree and had received comprehensive training in qualitative research methodology, and had enough relevant experience in multiple qualitative research studies. In the meantime, the first author had enough experience in the clinical nursing of lung cancer patients, and was located in the department of the interviewees during their participant hospitalization. To ensure creditability of the study, the author had care for the participants during their hospitalization and had a long‐term engagement with the subjects and established a proper relationship. In the interest of consistency, the lead author conducted all interviews. Before the interviews, the first researcher contacted face to face with the patients when they were free during the postoperative period, and asking if they were willing to talk about their symptoms and psychological experiences of lung cancer convalescence after radical lobectomy. Then the researcher contacted the interested potential participants and given a written participant information sheet explaining the study, and invited them to participate in an in‐person interview. After a candidate agreed to participate, the first researcher briefly informed him or her about the study, the related objectives and the research method; each candidate then signed a letter of informed consent to ensure that the study would not have any impact on the future treatment of the diseases present among the interviewees. Then, the researcher scheduled an interview meeting at a time and interview location (such as a meeting room) that was quiet, well‐lit and allowed for undisturbed conversation. No other individuals were present except the participant and the interviewer at the time of the interview. The interview content was appropriately adjusted according to the interview situation so that the interviewees could talk freely and deeply express their thoughts, feelings and experiences. The techniques of questioning, repetition, and responding were adopted for in‐depth communication without interruption or judgment. The whole process was combined with audio recording to record the changes in the interviewees' expressions and behaviors.

The interview script was preliminarily formulated by referring to a large number of relevant studies and combining the findings with the research purpose. The research group discussed and preinterviewed three participants who were in the convalescence stage after radical lung cancer surgery who met the inclusion criteria, then the group finally determined the formal interview script. These three cases were not included in the study. The final interview questions included the following: What were the symptoms and manifestations of your body during postoperative rehabilitation? This question was followed by the query “What symptoms were most troubling to you?” The researcher also asked, “What was the psychological impact on yourself before and after the operation,” “What was the impact on your family before and after the surgery?,” “How did your social and professional life change before and after the surgery?,” and “How have you dealt with these changes?”

During the interviews, the interviewer asked questions that arose about the interview data by asking appropriate questions, such as “Can you tell me more about this?” In general, the purpose of asking follow‐up questions is to clarify relevant aspects of the subject. All interviews were conducted in Chinese by the first researcher and audio recorded. The duration of each interview ranged from 20 to 60 min, and the average duration was 40 min. The names of participants were replaced by numbers, and the interview data were kept strictly confidential. Field notes were written after each interview, and the interviews were transcribed in Chinese immediately.

Sociodemographic information and most of the clinical information were collected from the participants. Data collection continued until the information collected reached the saturation point and no new themes or information appeared from the interviews.^21^

### 2.4 Data analysis

Within 24 h after the interview, the first researcher (Julan Xiao) transcribed the recorded content and numbered the verbatim manuscript in the order of the interview times,^22^ that is, from P01, P02, P03…P18, encoding each transcript and interview transcript in turn. Another researcher (Yi Wen, who worked as an oncology nurse for many years) in the research group checked the recorded and transcribed interview texts to ensure the completeness and accuracy of the content, which was analyzed by Colaizzi's seven‐step method of phenomenology.^23^ Two researchers (Julan Xiao and Yi Wen) carefully read and analyzed the written data, coded and summarized the meaningful statements that repeatedly appeared and were consistent with the patient's symptom experiences and psychological feelings during the rehabilitation period, distinguished similar views, and finally, extracted the themes and returned them to the interviewees for verification to verify the authenticity and reliability of the data. The themes and subthemes were collected in a Microsoft Excel spreadsheet, with supporting descriptions, and quotes.

### 2.5 Rigor

To ensure the rigor and trustworthiness of the study, several methods were used. This study was reported in accordance with the COREQ checklist. The dependability, credibility, transferability, and confirmability of the data were evaluated.^24^ First, to enhance the dependability of the research, any disagreement pertaining to the design, methods, data analysis, and results were all discussed in the research group until a consensus was reached. Second, two researchers independently coded all interview transcripts and analyzed them repeatedly to reduce the level of bias in the text analysis. The research group discussed and confirmed the major themes and subthemes and reached consensus regarding their organization and relationships. Third, the researcher provided encoded interviews to the subjects to ensure that the extracted codes matched their feelings and experiences. Moreover, regarding the transferability of the study, the researcher described the research methods in detail to provide a reference for future research.

### 2.6 Ethics considerations

This study was approved by the Ethics Committee of Shenzhen People's Hospital (Approval No. LL‐KY‐2022004‐01). Before the interviews, all participants were informed of the purpose of the interviews and required to sign consent forms that permitted them to withdraw from the study at any moment.

## 3 RESULTS

A total of 18 patients in the convalescent stage of lung cancer took part in the study. Respondents' mean (SD) age was 55.06 ± 10.22 years. Demographic information is shown in Table 1. The participants consisted of more men (11 men [61.1%]) than women (7 women [38.9%]). Most respondents had less than a junior college education (11 respondents [61.1%]). Lung cancer stage data showed 8 respondents (44.4%) who had stage II cancer and 6 respondents (33.33%) who had stage III cancer; 12 of 18 participants (66.7%) had adenocarcinoma cancer at diagnosis. Most of the respondents were diagnosed with lung cancer less than 6 months prior (*n* = 12). The participants were all receiving operative treatment.

**TABLE 1:** Demographic information and clinical characteristics of the participants (*n* = 18).

ID code	Sex	Age (years)	Education states	Occupation	Family monthly income per capita (Chinese yuan)	Medical burden	Type of pathology	Stage of cancer	Time since confirmed cancer diagnosis (months)
P01	Male	50	Master	Civil service	10,000	No burden at all	Adenocarcinoma	II	6
P02	Male	42	Senior high school	Worker	5000	Certain burden	Squamous cell carcinomas	II	2
P03	Male	58	Senior high school	Worker	3000	Heavy burden	Squamous cell carcinomas	III	12
P04	Male	61	Senior high school	Retired	3000	Certain burden	Adenocarcinoma	I	3
P05	Female	34	Undergraduate	Self‐employed	10,000	No burden at all	Adenocarcinoma	I	12
P06	Male	52	Junior College	Worker	10,000	Basically no burden	Adenocarcinoma	II	8
P07	Male	56	Primary school	Farmer	2000	Heavy burden	Squamous cell carcinomas	II	6
P08	Female	47	Undergraduate	Clerk	8000	Basically no burden	Adenocarcinoma	I	2
P09	Female	54	Primary school	Farmer	2000	Heavy burden	Adenocarcinoma	III	6
P10	Female	57	Undergraduate	Retired	5000	Certain burden	Adenocarcinoma	II	3
P11	Male	66	Senior high school	Retired	7000	No burden at all	Squamous cell carcinomas	II	5
P12	Male	35	Senior high school	Worker	10,000	Basically no burden	Adenocarcinoma	I	12
P13	Female	67	Primary school	Retired	6000	Basically no burden	Squamous cell carcinomas	III	5
P14	Male	63	Senior high school	Retired	5000	Certain burden	Squamous cell carcinomas	III	3
P15	Male	58	Junior College	Clerk	10,000	Basically no burden	Adenocarcinoma	II	3
P16	Female	58	Junior College	Retired	5000	Certain burden	Adenocarcinoma	III	12
P17	Male	65	Junior school	Retired	6000	Basically no burden	Adenocarcinoma	III	3
P18	Female	68	Primary school	Retired	2000	Certain burden	Adenocarcinoma	II	2

From the analysis of interviews, four themes emerged from the data related to patients' symptom experiences and psychological feelings: (1) physiological dimensions; (2) psychological dimensions; (3) family dimensions; and (4) social dimensions. Themes and categories are presented in Table 2. In the following part, excerpts from individual interviews are presented under the name of each theme.

**TABLE 2:** Summary of study findings (*n* = 18).

Categories	Subcategories
Physiological dimensions	Feelings of fatigue or tiredness
	Cough or phlegm
	Chest tightness and shortness of breath
	Daily activities were affected
	Sleep disturbance
	Lack of appetite
Psychological dimensions	Negative emotion
	Fear of cancer recurrence
	Learn to accept the reality
	Strengthened faith and hope
Family dimensions	Heavy economic burden
	Perceived family care
	Improved health management behavior
Social dimensions	Perceived support of medical staff
	Decreased sense of social worth and self‐identity
	Changes in social and working styles

### 3.1 Theme 1: Physiological dimensions

#### 3.1.1 Feelings of fatigue or tiredness

As a result of the disease and the radical lung resection, the patients' body tolerance was poor, and a majority of the participants were prone to fatigue or tiredness. They recalled that just a little walking or activity engagement would make them feel low on energy.

Walking is very hard, plus doing housework; for example, when I clean the floor at home with a towel, I am very tired. (Participant 09)

I still go out for a walk every day, but my legs are weak, and my level of strength is a little low. (Participant 14)

#### 3.1.2 Cough or phlegm

Most patients with lung cancer were prone to a persistent cough after lobectomy and lymph node dissection. They complained that the symptom that bothered them most was their cough. They also worried and became depressed thinking that if they coughed too much, the wound at the surgical site would crack.

A little exercise and then I cough—gasp; I dare not do strenuous exercise. (Participant 06)

After the operation, I still coughed for two months, with a lot of white sputum; I needed sputum stimulation to cough it out (frown). (Participant 16)

#### 3.1.3 Chest tightness and shortness of breath

Most patients voiced that they felt chest tightness and shortness of breath. Two patients said what bothered them most was the problem of breathing. They were aware that it was hard to walk or talk, and they could not breathe when they talked or exercised. Due to the lobectomy, the integrity of their lungs was affected, resulting in respiratory dysfunction of the patients, which manifested as chest tightness and asthma.

I had a part of my lung removed, so I would gasp when I went out to exercise. The doctor said I should recover 80% of my normal respiratory function within 3 months of the surgery. (Participant 13)

While walking I would wheeze; suddenly there was a shortness of breath, sometimes at a certain point I would feel out of breath, but I could not express it; it felt very painful. (Participant 15)

#### 3.1.4 Daily activities were affected

Some patients reported postoperative respiratory dysfunction and decreased lung capacity, which caused difficulties in daily activities. They explained that if they went out for activities, they would feel a little tired after a short walk. Thus, their daily activities suffered negative impacts.

Before the disease, I was alive and kicking and could do anything. Because I had a lot of surgical resection, it had a great impact on my respiratory function. If I exercised a little bit, I would be out of breath and cough. (Participant 06)

Mainly physical changes; my body was not strong enough to exercise in a big way. (Participant 15)

#### 3.1.5 Sleep disturbance

Respondents mentioned having difficulty falling asleep due to recurrent coughing at night, which affected their sleep and QOL. They complained that this disease disturbed them and said that they coughed all day and all night, which made it difficult for them to sleep at night.

Sometimes lying down at night to sleep well, a sudden cough would make me very uncomfortable; I began to have spasms of phlegm, and this would always make me not sleep well. (Participant 16)

#### 3.1.6 Lack of appetite

Due to the invasion of cancer cells, the growth of lung tumors consumes various vitamins in the body, resulting in a lack of nutrients and long‐term and repeated loss of appetite, resulting in malnutrition, body mass decline and other physiological problems. Many patients recognized that owing to this disease, they often cannot eat or sleep well. One participant felt that she was often unable to eat and experienced a loss of appetite.

Every now and then, when I have phlegm, I have to clean it up, and then I have no appetite when I come back to the table. (Participant 13)

### 3.2 Theme 2: Psychological dimensions

#### 3.2.1 Negative emotion

As a major stress event, the diagnosis and treatment of lung cancer can cause serious trauma to patients' physical and mental health, resulting in negative emotions such as hopelessness, unfairness, sadness, fear, grief, and collapse. Owing to incurable cancer and worsening physical conditions, helplessness and hopelessness were found to be the most common negative emotions of patients. In addition, they stated that they were too young to get cancer and could not accept the fact that the pathology of the lung nodule was confirmed as lung cancer. Several participants avoided using the word “cancer,” believing that the mention of the disease may invoke it.

I didn't think it was cancer. It was very young to get cancer since I was less than 50 years old. I was a little depressed at that time; how could it be cancer? (Participant 01)

For a period of time, my mood would be very low, just like a balloon, the state of being half in the air; I would certainly have negative emotions, then suddenly feel like I was collapsing. (Participant 06)

#### 3.2.2 Fear of cancer recurrence (FCR)

Although some patients recovered well after the operation, the convalescent patients were still worried about the prognosis and recurrence of the disease. They were worried about relapse during the postoperative recovery period.

My biggest concern was how high the survival rate of this disease was and whether it would recur. (Participant 07)

Following the changes in my condition after the completely radical treatment, relapse was my top worry. (Participant 12)

#### 3.2.3 Learn to accept reality

Suffering from lung cancer was a heavy blow to the patients. After constant adjustments of their mentality, some patients gradually learned to accept reality. They realized that they thought too much was useless, and they could not change it; regardless, they had to face up to it.

It is impossible not to get sick in one's life. We should face things positively and avoid complaining about them. If there is a problem with my physical condition, I need to sum up my living habits and make appropriate adjustments to my anxiety level in all aspects. (Participant 03)

Since I have this disease, I can only receive treatment. As the saying goes, which comes first, tomorrow or an accident? I would say accident (smile). (Participant 06)

#### 3.2.4 Strengthen faith and hope

For cancer patients, faith and hope are useful psychological resources that can help alleviate patients' negative emotions and strengthen their belief in overcoming the disease in the process of fighting against the disease. Many patients described that they were acceptive of their illness and had adapted to the lung cancer patient's role. They also reported that they trust that the current level of medical treatment and services will enable them to overcome their diseases.

Mentally, all I can do is actively cooperate with treatment and have surgery as early as possible (positive face). (Participant 02)

As long as I have good treatment, I will be better. The current difficulty is temporary, this year, I plan to cure the disease and carry over the challenge. Next year will certainly be better than this year. (Participant 09)

### 3.3 Theme 3: Family dimensions

#### 3.3.1 Heavy economic burden

A small number of respondents said that the high cost of lung cancer treatment was a heavy economic burden on them and led to them overly worry about their family's economic situation. In general, due to the reality that the treatment cycle is long and expensive, the sick period conflicts with working hours, and family income decreases. Thus, a patient's whole family comes under enormous financial pressure.

The economic pressure has a great impact on me. Now I have to borrow money from relatives and friends everywhere to go to doctors, and we became a poor family after my illness (choked up and crying). (Participant 03)

The difficulty is the economics. I came from the countryside, so I was the only one to earn money. After I got sick, I could not do anything, and I did not know when I could go back to work. (Participant 07)

#### 3.3.2 Perceived family care

A majority of patients perceived receiving financial support, emotional support or family care support from spouses or children that promoted patients' physical and mental health through family encouragement and psychological support. They explained that their family took good care of them, especially when they were ill; furthermore, their family was very united in taking care of them, which made them feel happy.

Since they were worried about my illness, my family told me not to work. I was in a bad mood and would turn to my husband in anger, but my husband was very tolerant and took care of me. (Participant 09)

My children took care of me when I was hospitalized, when I had surgery and when I was discharged. They took good care of me very carefully…with smile on their face. (Participant 17)

#### 3.3.3 Improved health management behavior

After reflecting on the attribution of disease, a few patients realized the importance of health, constantly improved their health management behavior, paid more attention to physical health, exercised and cultivated a healthy lifestyle. They recognized that a variety of proper exercise programs, careful diet, and other work that they could do would give their body some improved pleasure.

The key is to change your lifestyle. For example, I learned that this lung adenocarcinoma was related to my irregular lifestyle, staying up late and smoking. Therefore, changing our lifestyle is the most critical thing. (Participant 01)

This operation has made me pay more attention to health management, and I will get physical examinations in a more timely manner in the future. I also told my family to go get a physical examination every year; in the meanwhile, we should pay more attention to these physical examinations. (Participant 05)

### 3.4 Theme 4: Social dimensions

#### 3.4.1 Perceived support of medical staff

Some patients said that they received care and support from medical staff during this period, which increased their confidence in overcoming the disease. They stated that the medical professionals were warm and did everything they could to do to meet patients' needs.

The doctor was very responsible, and the service attitude and service level were good. The doctors and nurses were very professional; they did what should be done well. (Participant 1)

With the encouragement and support of the doctors and nurses, I felt that tomorrow would be better. “Hopefully tomorrow, the future will be bright” (Participant 6)

I am very grateful to the doctors and nurses who care about their patients very much. They explained everything to me and arranged for me to be examined or treated for any discomfort. I did not have so much to worry about. (Participant 17)

#### 3.4.2 Decreased sense of social worth and self‐identity

The social function of patients with lung cancer after surgery was affected, and some patients had doubts about their sense of self‐worth and work ability. When lung cancer patients could no longer fulfill their family responsibilities, such as supporting their parents or educating their children, instead of being cared for, their sense of social worth and self‐identity decreased.

I can't work, and I can't help my son take care of the baby. I feel that I am a burden to the family. (Participant 9)

My illness puts a burden on my children, who have to pay the mortgage and my medical bills. My energy level also affects my children, who need time off for my hospital stay, surgery and hospital discharge to take care of me. (Participant 17)

#### 3.4.3 Changes in social and working styles

All patients indicated that they avoided social activities during the postoperative rehabilitation period and hardly had any social activities. In addition to the incomplete recovery of their physical condition, they were also afraid of being asked about their disease status.

I used to like to go out and dance every day, but since the surgery, I have not been able to dance with my friends, for fear that people will ask me about this disease. (Participant 13)

I didn't tell my friends or relatives that I had lung cancer because it is not a good thing to have. I was afraid they would worry about it, and I don't want anyone to know. (Participant 14)

Some respondents felt that their jobs had been affected by their illness and that their jobs and the nature of their work had changed.

In the future, we should adjust our way of work; work can slow down. First take good care of our body, give yourself some rhythm, and don't be so tired. (Participant 1)

I haven't reached retirement age yet. I might consider early retirement or some kind of work arrangement to keep myself young. (Participant 6)

My future work will be step by step; if I can go to work, I will work well, but take it easy; take care of our health is the most important thing. (Participant 9)

## 4 DISCUSSION

This study is significant in that it reveals findings that enrich the literature by addressing the symptoms and psychological experiences of convalescent patients with lung cancer after radical lobectomy in China, presents data for the development of cancer survival care models, and provides health professionals with a culturally specific perspective. The current research deeply explores the symptoms and psychological experiences of lung cancer patients with regard to physiological, psychological, family and social dimensions. Overall, these experiences provide a holistic view of the multifaceted issues surrounding lung cancer convalescence after radical lobectomy.

Similar to previous studies^25^, ^26^ in our current study, we observe that after radical lung cancer surgery, patients suffer from various symptoms, such as fatigue, cough, and respiratory dysfunction, which seriously compromise the QOL and affect the postoperative rehabilitation of patients. For early‐ and middle‐stage lung cancer, radical lobectomy, and peripheral lymph node dissection are the preferred treatment methods. Due to the particularity of surgical methods, patients are prone to physical decline, lymphedema, and impaired respiratory function after surgery.^27^ Cough is one of the common complications after radical lung cancer surgery. However, while moderate coughing after surgery is helpful to promote sputum excretion and lung reexpansion and reduce lung infection, excessive pathological coughing will cause a series of complications, such as fatigue and sleep disorders, thereby prolonging the postoperative recovery process.^28^ As in the study of Rodrigues et al.^29^ sleep disturbance is also an important predictor of fatigue. Due to the invasion of the tumor into the lung, the original complete anatomical structure of the chest is changed after radical lung cancer surgery, resulting in respiratory dysfunction. Loss of appetite in patients with lung cancer easily causes malnutrition and weight loss, which can directly affect their QOL. Combined with symptom management theory,^30^ one of the important therapeutic goals is to pay attention to the postoperative symptoms, strengthen the symptom management, prevent and reduce the postoperative complications, and improve the QOL of lung cancer patients.^31^ In conclusion, it is suggested that medical professionals should therefore attach great importance to the symptom management of patients after radical lung cancer surgery, engage in timely and accurate professional evaluation, strengthen the health education of related symptoms, guide patients and their families to carry out effective symptom management, and help patients reduce their symptoms and level of trouble. At the same time, regular assessment and guidance of symptom management should be carried out in combination with various approaches, such as specialized pulmonary rehabilitation clinics, to meet patients' diversified care needs.

Lung cancer has a high fatality rate and seriously damages one's health. Compared with patients with other types of cancer, the symptoms of patients with lung cancer are characterized by rapid occurrence, heavy severity, large energy consumption, long duration, and seriously compromised QOL,^32^ all of which easily cause patients to have adverse psychological experiences.^30^ Our study found that patients with lung cancer experience hopelessness, unfairness, sadness, fear, grief, depression, and other negative emotions, and they attribute the cause of each negative situation to lung cancer, which may be related to FCR, metastasis, routine controls, financial, and symptom burden. Concern about FCR is considered to be the longest lasting effect of cancer and has been widely addressed in the literature.^33^, ^34^ Statistically, the prevalence of FCR is 39%–97%,^35^ as it is one of the most common psychological burdens experienced by cancer patients.^36^ The literature illustrates that depression is more severe among lung cancer patients than among patients with other types of cancer.^7^ In addition, the symptoms of lung cancer patients can also undoubtedly cause distress to their physical and mental state and seriously compromise their QOL.^7^ All this indicates that medical professionals should make concerted efforts to understand the psychological experiences of lung cancer patients and respond to them appropriately. Therefore, we believe that it is essential for the health professional team, including families in care, to concentrate on patients' psychological health, as well as their physical rehabilitation.

Combined with the stress process model, life events can lead to individual stress responses; the diagnosis of lung cancer itself becomes a serious stress event, which poses a threat to the mental health of patients. Our study suggests that hospitals should set up special nursing psychological counseling clinics after lung cancer surgery, carry out one‐on‐one psychological counseling, conduct positive psychological adjustment, guide patients to establish a good psychological stress mechanism, and reduce negative emotions.

In this study, it is revealed that through constant adjustment, patients learn to accept the reality, face it calmly, and have firm faith and hope that they will overcome cancer, which cannot be separated from diversified family care, and social support from medical staff. Postoperative patients with lung carcinoma experience significant changes in their lives, especially in regard to their family and social relationships and self‐esteem. Family and close circle support has a strong influence on increasing one's resilience levels and adapting to a new life after lung cancer. The literature highlights^37^ that doctors provide professional information support so that patients with lung cancer can fully understand the nature of the disease; the treatment, management, and control of lung cancer the side effects of drugs; and the prognosis, as well as information about a healthy lifestyle and daily living activities, which is similar to findings of the present study. At present, the foreign nursing model of lung cancer supportive care is mainly the electronic health system led by nurses specialized in lung cancer.^38^ The results of our study and existing studies reveal that understanding the needs of cancer patients helps health professionals provide appropriate counseling services for patients. Postoperative patients with lung carcinoma need to be supported psychosocially by different health care providers, such as doctors, nurses, and psychologists.

In addition, our study finds that some patients with lung cancer can exert their own positive energy, actively cope with and adjust to the related changes, understand the meaning of life based on their reflections about their own lung cancer, rebuild a healthier and more scientific lifestyle, and gradually pay attention to their own medical care and the health of their relatives. However, it is also found in the interviews that the social style and work nature of lung cancer patients change after surgery. Some patients hide their illness from relatives and friends, and their sense of self‐identity and social value decreases in the process of returning to society. While receiving treatment and rehabilitation, postoperative patients with lung cancer experience an increase in feelings and thoughts, such as role confusion, not being able to support their parents and children, and guilt. There is evidence^11^ that there is a link between improving social support and improving the QOL of lung cancer patients; thus, increasing the level of social support is one of the most important factors in improving the QOL of lung cancer patients. Social interaction can lead to positive experiences that make patients perceive life as being more predictable and stable, and patients are more likely to comply with medical recommendations and participate more in the treatment process.^32^ In addition, peer support is beneficial for postoperative patients with lung carcinoma^39^; in addition, the peer support system can be used to overcome time and geographical limitations, create a friendly environment for cancer friends, and increase the social participation of patients to give full play to the function of the peer support system.^40^ For this reason, we recommend that peer support be taken into account by the health care team.

### 4.1 Study limitations

There are several limitations to this study. First, the presentation of data based on a single culture may limit the generalizability of the findings. Second, the results were derived from lung cancer patients' self‐reports, which means that there is possible subjectivity and bias in this study. Third, while focusing on the experiences of postoperative patients with lung carcinoma, no distinction was made; thus, the experiences are presented in general terms. Despite these limitations, this study yields valuable insights into the symptoms and psychological experiences of postoperative patients with lung carcinoma in China. Another strength of the study is that we used the COREQ checklist for methodological rigor and transparency while conducting and reporting the study.

## 5 CONCLUSIONS

In conclusion, our research provides insights into the perspectives of patients in the convalescence period after undergoing radical lung cancer surgery with regard to their symptoms and psychological experiences. Our findings suggest that patients in the radical lung cancer postoperative rehabilitation period suffer from a certain degree of symptom distress and negative psychological experiences, all of which seriously compromise their QOL. With regard to patients with radical lung cancer, medical professionals should carry out positive and effective professional evaluations and symptom management in a timely manner, plan to provide targeted interventions and psychological support, strengthen and improve the continuity of nursing care in the postoperative rehabilitation period, help improve their QOL, and rebuild their confidence level, with the aim of returning to their family and society as soon as possible.

## AUTHOR CONTRIBUTIONS

**Julan Xiao:** Conceptualization (lead); data curation (lead); formal analysis (lead); investigation (lead); methodology (lead); project administration (lead); resources (lead); writing – original draft (lead); writing – review and editing (lead). **Yueming Peng:** Conceptualization (supporting); data curation (supporting); formal analysis (supporting); methodology (supporting); writing – review and editing (supporting). **Yun Li:** Data curation (supporting); writing – review and editing (supporting). **FengQing Ye:** Data curation (supporting); writing – review and editing (supporting). **Zhixong Zeng:** Data curation (supporting); writing – review and editing (supporting). **XiaoXu Lin:** Data curation (supporting); writing – review and editing (supporting). **Yanheng Xie:** Writing – review and editing (supporting). **Sijiao Cheng:** Writing – review and editing (supporting). **Yi Wen:** Conceptualization (supporting); data curation (supporting); formal analysis (supporting); investigation (supporting); methodology (supporting); project administration (supporting); resources (supporting); supervision (supporting); writing – review and editing (supporting). **Weixiang Luo:** Conceptualization (supporting); data curation (supporting); formal analysis (supporting); investigation (supporting); methodology (supporting); project administration (supporting); resources (supporting); supervision (supporting); writing – original draft (supporting); writing – review and editing (supporting).

## FUNDING INFORMATION

This study was supported by the Shenzhen Peoples' Hospital and Young and Middle‐aged Research Fund Project (grant number SYHL2021‐N0006), Shenzhen Health Economics Society Research Fund Project (grant number 202336) and Nursing discipline research topic of Chinese Medical Association journal (grant number CMAPH‐NRG2022042).

## CONFLICT OF INTEREST STATEMENT

The authors declare no potential conflict of interest with respect to the research, authorship or publication of this article.

## Data Availability

The data underlying this article will be shared on reasonable request to the corresponding author.
